# Aldose Reductase, Protein Glycation Inhibitory and Antioxidant of Peruvian Medicinal Plants: The Case of *Tanacetum parthenium* L. and Its Constituents

**DOI:** 10.3390/molecules24102010

**Published:** 2019-05-25

**Authors:** Seung Hwan Hwang, Hyun-Yong Kim, Yanymee N. Guillen Quispe, Zhiqiang Wang, Guanglei Zuo, Soon Sung Lim

**Affiliations:** 1 Department of Food Science and Nutrition, Hallym University, 1 Hallymdeahak-gil, Chuncheon 24252, Korea; isohsh@gmail.com (S.H.H.); khy9514@nate.com (H.-Y.K.); guangleizuo@foxmail.com (G.Z.); 2 Department of Molecular Medicine and Biopharmaceutical Sciences, Graduate School of Convergence Science and Technology, Seoul National University, Seoul 151742, Korea; estreyany@gmail.com; 3 College of Public Health, Hebei University, Baoding 071002, China; wangzq2017@hbu.edu.cn; 4 Institute of Korean Nutrition, Hallym University, 1 Hallymdeahak-gil, Chuncheon 24252, Korea; 5 Institute of Natural Medicine, Hallym University, 1 Hallymdeahak-gil, Chuncheon 24252, Korea

**Keywords:** ultrafiltration, DPPH-HPLC, aldose reductase, *Tanacetum parthenium* (L.) Schultz-Bip, sorbitol accumulation

## Abstract

Diabetes complications, including peripheral neuropathy, cataracts, impaired wound healing, vascular damage, arterial wall stiffening and retinopathy diseases, are among the most predominant health problems facing the world’s population today. The 22 Peruvian plant extracts were screened for their potential inhibitory activity against rat lens aldose reductase (RLAR) and DPPH radical scavenging. Among them, we have found that *Tanacetum parthenium* L. (TP) has the RLAR, AGEs and DPPH radical scavenging activities. We used for screening of active components in TP against RLAR and DPPH for the first time by ultrafiltration (UF) and DPPH. Compounds in TP were isolated by Sephadex column chromatography and their structures were established by MS and NMR spectroscopic analyses. Among the isolated compounds, ferulic acid, apigenin, luteolin-7-*O*-glucoside, luteolin, chrysosplenol, and kaempferol showed potent inhibition with IC_50_ values of 1.11–3.20 and 6.44–16.23 μM for RLAR and DPPH radical scavenging. Furthermore, these compounds suppressed sorbitol accumulation in rat lenses and ferulic acid, luteolin-7-*O*-glucoside, and luteolin have AGEs inhibitory activities with IC_50_ values of 3.43–6.73 μM. In summary, our study provides interesting plants for further study with respect to the treatment and prevention of diabetic complication of Peruvian plant and can provide the scientific base of the traditional uses.

## 1. Introduction

Aldose reductase (AR: EC 1.1.1.21) is the rate-limiting enzyme of the polyol pathway. AR catalyzes the conversion of glucose to sorbitol, and sorbitol dehydrogenase the second enzyme of the pathway-further converts sorbitol into fructose [1]. AR inhibitors (ARIs) have received considerable attention because of the proposed involvement of AR in the pathophysiology of diabetic complications, including cataracts [2]. AR-catalyzed formation of sorbitol has been observed in a number of tissues; in diabetes mellitus, increased sorbitol generated through the polyol pathway does not readily diffuse across cell membranes, and intracellular accumulation of sorbitol has been implicated in chronic complications of diabetes, such as cataracts, neuropathy, and retinopathy [3].

Glycation is a nonenzymatic browning reaction caused by an amino-carbonyl reaction between a reducing sugar and an amino group of a protein or lipid. The nonenzymatic reaction leads to chemical modifications of tissue proteins, called advanced glycation end products (AGEs), resulting in functional disturbances of the proteins [4]. In addition, both diabetes and aging are associated with accumulation of AGEs in tissues, increased oxidative stress, and decline in antioxidant status. The formation and accumulation of AGEs in many different cell types affect the extracellular and intracellular structure and function by inducing oxidative stress [5]. The complex, fluorescent AGE molecules formed during the Maillard reaction can lead to protein cross-linking and contribute to the development and progression of diabetic complications, such as peripheral neuropathy, cataracts, impaired wound healing, vascular damage, arterial wall stiffening, and decreased myocardial compliance [6].

Conventional bioassay-guided fractionation is widely used to discover new bioactive compounds, but it is a time-consuming, labor-intensive, and low-efficiency strategy [7]. However, 2,2-diphenyl-1-picrylhydrazyl (DPPH) radical and ultrafiltration high-performance liquid chromatography (HPLC) methods based on the theory of ligand–enzyme interactions have proved to be simple, efficient, and high-throughput methods, which show great potential for applications in the rapid screening of bioactive compounds from complex natural products [8,9]. In a previous study, an effective strategy to identify active components from natural products by DPPH radical and ultrafiltration HPLC was used to facilitate screening assays [10,11].

Many researchers have been trying to find a safe, potent and non-toxic diabetic complication drug and functional food source from natural products. In this study, to better understand the functional properties of Peruvian plants and provide evidence for the development of functional food materials from Peruvian plants, RLAR inhibition and antioxidant activities of 22 selected Peruvian plants were evaluated. In addition, *Tanacetum parthenium* L. (LNP-23, TP), which is a member of the plant family ASTERACEAE widely distributed in South America, showed the highest AR inhibition and considerable antioxidant effects. Therefore, four sets of experiments were performed to isolate and identify the active components from TP and to determine their inhibitory activities. First, ultrafiltration and DPPH-HPLC were used for screening of active components in TP against RLAR and DPPH for the first time. Second, the peaks of the bioactive components were identified using two HPLC methods, and the bioactive compounds were separated on a Sephadex LH-20 column. Third, the inhibitory activities of the isolated compounds toward RLAR, advanced glycation, and DPPH radical scavenging were investigated to evaluate their use in the treatment of diabetic complications. Finally, the ability of the major compounds showing activity against RLAR to decrease sorbitol accumulation in rat lenses under ex vivo high-sorbitol conditions was evaluated and the structural simulation of the complex was confirmed.

## 2. Results

### 2.1. Evaluation of Rat Lens Aldose Reductase and DPPH Radical Scavenging of 22 Peruvian Plant Extract

Most related research in Peru has been conducted in the Amazon and only a few studies have in La Libertad. In the present study, 22 Peruvian plants were collected from the department of La Libertad and the catalogue number of each plant is provided in Table 1.

Among them, four belonged to the FABACEAE family, two to the LAMIACEAE, POACEAE, ASTERACEAE and SOLANACEAE family, and other ten families. The crude extracts of 22 plants of various families were prepared using 70% MeOH and their yields were from 1.95% to 50.94%, of which *Capsicum baccatum* (LNP-46) had the highest yield. The inhibitory activities of 22 Peruvian plant extracts were investigated on RLAR and DPPH radical scavenging, and the results are reported in Table 1. Among the 22 Peruvian plant extracts, only four extracts, namely *Otholobium mexicanum* (L. f.) J.W. Grimes (LNP-3), TP (LNP-23), *Sambucus peruviana* H. B. K (LNP-24), and *Eleocharis albibracteata* Nees and Meyen ex Kunth (LNP-28), showed high RLAR inhibition (over 50%) at a concentration of 10 μg/mL. Significant inhibition of RLAR (up to 61.10%) was observed with TP (LNP-23). Among the 22 Peruvian plant extracts investigated, five extracts, namely *Cheilanthes pruinata* Kaulf. (LNP-1), *Clinopodium pulchellum* (Kunth) *Govaerts* (LNP-15), TP (LNP-23), LNP-24, and *Desmodium molliculum* (Kunth) DC. (LNP-80), showed relatively high antioxidant capacities with inhibition (%) ranging from 52.77–89.97% at a concentration of 75 μg/mL. TP (LNP-23), in particular, showed inhibitory activity against DPPH radical scavenging, with 88.58% inhibition. Moreover, TP showed AGE formation inhibition of 61.01% at a concentration of 200 μg/mL (Table 2). The 22 Peruvian plant extracts were prepared in 70% MeOH and their yields were from 1.95%–41.95%, among which TP (LNP-23) showed a medium yield, as listed in Table 1. For these reasons, we focused on the isolation of aldose reductase inhibitors from TP (LNP-23) in this study.

### 2.2. Ultrafiltration High-performance Liquid Chromatography Screening of AR Inhibitors in Tanacetum parthenium L

**Figure 1:**
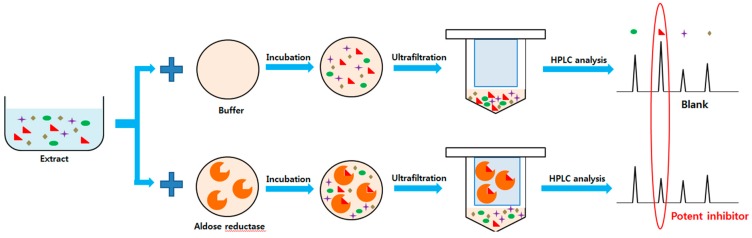
HPLC-based strategy for screening aldose reductase inhibitors from natural product extracts.

The ultrafiltration HPLC method can be used for rapid screening of bioactive components from natural products without sample pretreatment [12]. TP was incubated with HRAR. Components with inhibitory activity for AR could be separated from unbound components by the ultrafiltration membrane after binding with HRAR (Figure 1).

**Figure 2:**
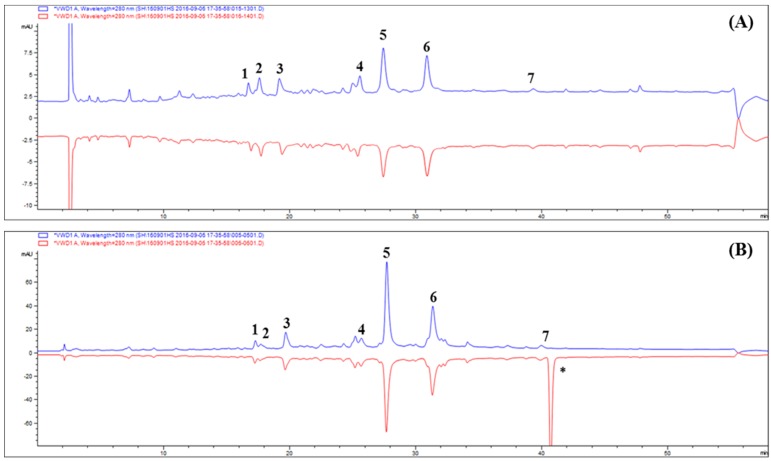
Screening of aldose reductase and antioxidant inhibitors in *Tanacetum parthenium* L. using by ultrafiltration of HRAR (**A**) and DPPH-HPLC (**B**) at 254 nm. Blue line: before reaction without HRAR and DPPH; red line: after reaction with HRAR and DPPH, respectively.

The results suggested that six components combined with active HRAR and could be considered as major ARIs in TP. According to the results in Figure 2A, although the target compounds are still detected in the eluent of deactivated HRAR (blue line), the decrease in peak area proves that the combination between HRAR and inhibitor is achieved through specific binding (red line).

Therefore, seven ARIs were preliminarily identified through ultrafiltration HPLC. The results demonstrate that compound 4 possesses the greatest degree of total binding (TBD; 38.02%) followed by **1** (27.08%), **3** (26.58%), **5** (25.54%), **6** (24.29%), **2** (15.00%), and **7** (0%), which might be caused by their structures and concentrations (Table 3). Moreover, the complex mixture exhibited competitive relationships, and the binding degree in a complex mixture was sometimes different from that of a single component.

### 2.3. Screening Antioxidant Activity in Tanacetum parthenium L. by DPPH High-performance Liquid Chromatography Analysis

Figure 2 shows the DPPH-HPLC assay of TP, and DPPH reaction of TP in Figure 2B (red line) shows that the peak areas are obviously reduced relative to those before reaction (blue line). The peaks of compounds with potential antioxidant activities will be reduced or will disappear in the HPLC chromatogram after reaction with DPPH, whereas the peak areas for those without antioxidant activities will have almost no change [8]. The presented peak areas for compounds **1**–**7** are obviously reduced, and in this experiment, the DPPH reaction mechanism supports this conclusion. Therefore, compounds **1**–**7** in TP possess antioxidant activity, and compounds **1** and **4** are the major active compounds based on the relative peak areas in the HPLC chromatogram. The results demonstrate that compound **4** possesses the highest reduction of peak area (PAR; 29.36%) followed by **1** (23.53%), **3** (16.85%), **6** (13.97%), **2** (11.28%), **5** (7.19%), and **7** (0%), which might be caused by their structures and concentrations (Table 3).

### 2.4. Structural Determination of Isolated Compounds

**Figure 3:**
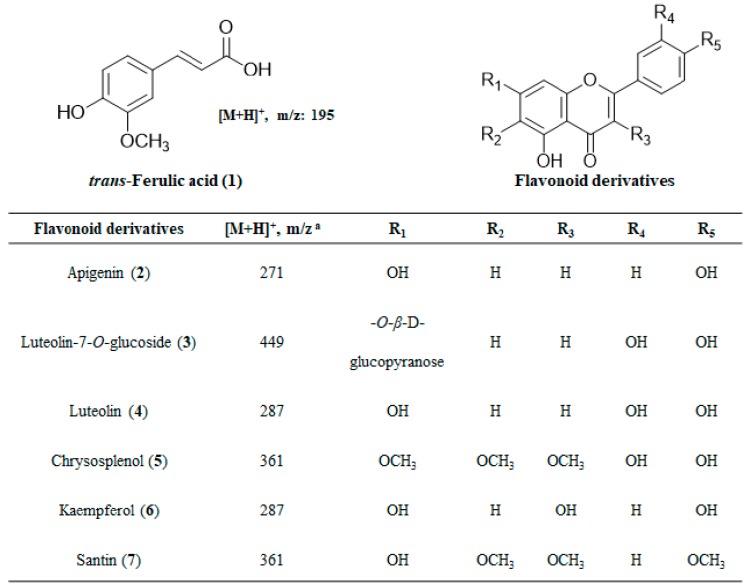
Chemical structures of compounds isolated from *Tanacetum parthenium* L. ^a^ The mass to charge ratio was obtained by Electron ionization-Mass (EI-MS) or Fast atom bombardment-Mass (FAB MS).

TP showed strong inhibitory activities on RLAR, AGEs, and DPPH radical scavenging activity (Table 2). Seven compounds were separated from TP by Sephadex LH-20 column chromatography. These compounds were identified by comparing ^1^H and ^13^C nuclear magnetic resonance (NMR) spectra and correlation NMR spectra such as COSY, HMBC and HSQC with previously reported data and by mass spectrometry. The compounds were ferulic acid (1, 96.21% purity), apigenin (2, 97.30% purity), luteolin-7-*O*-glucoside (3, 96.17% purity), luteolin (4, 94.33% purity), chrysosplenol (5, 93.17% purity), kaempferol (6, 98.01% purity), and santin (7, 91.97% purity) (Figure 3).

The inhibitory activities of TP isolated compounds against RLAR including sorbitol accumulation, AGE formation, and DPPH radical scavenging were evaluated to elucidate the biological activity [13,14,15,16].

### 2.5. Inhibitory Effects of the Isolated Compounds on Rat Lens Aldose Reductase

All the isolated constituents were examined for inhibitory activities against RLAR. TP showed high inhibitory activity against RLAR with an IC_50_ value of 8.04 μg/mL (Table 3). Compounds **1, 2**, and **5** exhibited RLAR inhibitory activities to varying degrees with IC_50_ values of 3.20, 1.97, and 1.92 μM, respectively, while compounds **3** (1.31 μM), **4** (1.76 μM), and **6** (1.11 μM) exerted stronger inhibitions than the quercetin (1.77 μM), which is commonly used for positive control. These results matched our predictions from ultrafiltration HPLC.

### 2.6. Inhibitory Activities on the Sorbitol Accumulation by Active Compounds

The accumulation of sorbitol in lens fiber cells increases the lens osmotic stress. AR-dependent synthesis of excess polyol has been implicated as one of the mechanisms causing diabetic cataracts [17]. Sorbitol accumulation in rat lens was found to be inhibited effectively by RLAR inhibitory compounds **1** (80.27%), **2** (87.07%), **3** (95.23%), **4** (91.83%), **5** (82.31%), and **6** (97.95%) at 5 μg/mL. The positive control (quercetin) inhibited sorbitol accumulation in rat lens by 85.71%, thereby reducing the sorbitol level in a culture medium containing a high glucose concentration (Table 4).

The results suggested that the components from TP can be used for the prevention and/or treatment of various diabetic complications by preventing the conversion of glucose to sorbitol. Moreover, the structures of flavonoids affected the RLAR inhibitory activities as well as the sorbitol accumulation effects.

### 2.7. DPPH Radical Scavenging Activity of the Isolated Compounds

The DPPH radical scavenging assay is the most popular spectrophotometric method for determining the antioxidant capacity of natural products because this chromogen radical can directly react with antioxidants. Additionally, DPPH radical dot-scavenging methods have been used to evaluate the antioxidant activities of compounds, due to the simple, rapid, sensitive, and reproducible procedures [18]. In this study, the DPPH radical-scavenging method was used to assess the potential radical-scavenging activities of TP, and the compounds **1**–**7** isolated from it. TP exhibited potent inhibitory activity against DPPH free radical scavenging activity (IC_50_ = 33.22 μg/mL) compared to the positive control L-ascorbic acid (IC_50_ = 6.02 μg/mL). The scavenging activities of compounds **1**–**7** and the positive control (L-ascorbic acid) on the DPPH radical decreased in the order: L-ascorbic acid > compounds **3 > 6 > 4 > 2 > 1 > 5 > 7**, having IC_50_ values (μM) of 3.41, 6.44, 8.32, 14.06, 16.23, over 25.0 > not inhibition, respectively (Table 3). The antioxidant activities of the isolated compounds closely matched the results of the DPPH-HPLC assay.

### 2.8. Inhibitory Effects of the Isolated Compounds on Advanced Glycation End Products Formation

The seven compounds isolated from TP were assessed for inhibitory activities towards the formation of AGEs by the glycation of BSA with methylglyoxal. TP showed an IC_50_ value below 163.71 μg/mL when compared with aminoguanidine (IC_50_ = 121.96 μg/mL) (Table 2). Polyphenols and flavonoids isolated from TP, which are the main metabolites of natural products, were also evaluated, and the results are summarized in Table 3. Among the tested compounds, compounds **2**, **5**, and **7** exhibited very little inhibitory activity towards the formation of AGEs with inhibition of 8.14%, 5.38%, and 13.15% at a concentration 20 μg/mL, therefore no further AGEs inhibitory IC_50_ values of these compounds were calculated as shown in Table 3. However, compounds **1**, **3**, and **4** showed inhibitory effects with IC_50_ values in the range of 3.43–6.73 μM, revealing that these compounds were more potent than aminoguanidine (IC_50_ = 110.55 μM), which was used as the positive control. Among these compounds, compound **3** showed the highest level of inhibition with an IC_50_ value of 3.43 μM, 32.23 times higher than aminoguanidine. On the other hand, AGEs results showed no significant relationship between the structure of the inhibitory compounds and their inhibitory activities. Recently, the inhibitory effect of flavonoids toward AGEs formation has been reported by Matsuda et al. [19]. In this study, compounds **3**, **4**, and **4** showed inhibition of AGEs formation for diabetic complication.

### 2.9. Interaction Analysis of Isolated Compounds with Aldose Reductase

**Figure 4:**
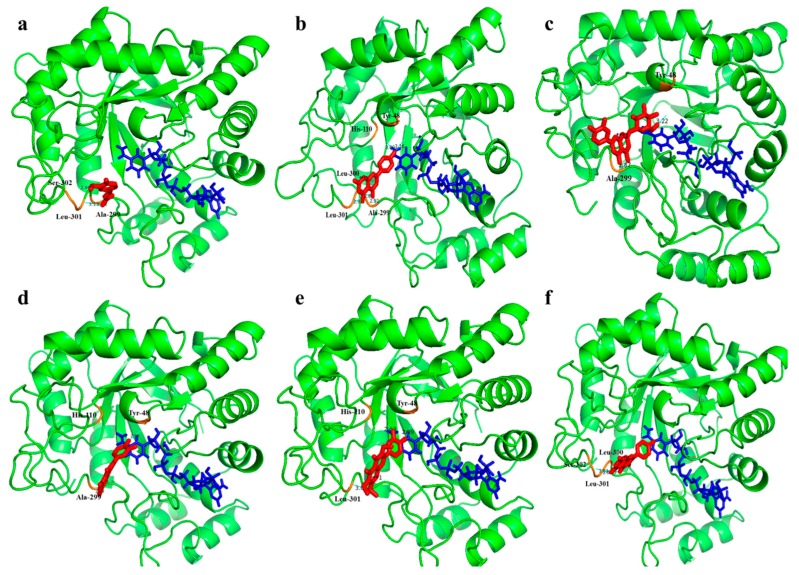
Docking models of ferulic acid (**a**), apigenin (**b**), luteolin-7-*O*-glucoside (**c**), luteolin (**d**), chrysosplenol (**e**), and kaempferol (**f**). The structure of aldose reductase is in green; the structures of the ligands are in red; the interactions of the residues with the ligands are shown in orange. (For interpretation of the references to color in this figure legend, the reader is referred to the web version of this article).

Docking interactions showed that the compounds **1–6**, isolated from TP, bind stably with AR (Figure 4).

Compound **1** binds to the active site of AR at Ala-299, Leu-301, and Ser-302 residues and compound **2** was binds at the Ala-299, Leu-300, Leu-301, His-110, and Tyr-48 residues. Similarly, compound **3–6** bind with Ala-299, Try-48, His-110, and Ser-302 residues on the active site of AR. All six compounds occupied the active site and interacted with the surrounding residues at different orientations. The molecular docking method can reveal the nature of ligand binding at the active site for various compounds. Our molecular docking simulation suggested that the strategy for screening AR inhibitor from natural products is reliable and can be used to distinguish the specific inhibitors from false positives.

## 3. Discussion

Repeated column chromatography with bioassay-guided fractionation is commonly used to find active components from natural products. However, this takes a long time, many solvents, and various conditions [20]. Therefore, many researchers have tried to develop selective, sensitive, and efficient technologies to screen and identify active components from natural products by using HPLC [21,22]. Previously, Zhou et al. reported that nine polyphenols isolated from *Radix astragali* were analyzed by using α-glucosidase by ultrafiltration HPLC [12]. In addition, components with inhibitory activities in *Glycyrrhiza uralensis* and *Polygonatum odoratum* were screened by tyrosinase and α-glucosidase ultrafiltration HPLC methods [23,24]. In addition, the inhibitory activity of these compounds was well matched with the results of the ultrafiltration HPLC assays. Therefore, the ultrafiltration method described is a very simple, straightforward, rapid, robust, and selective technology for discovering real bioactive components in TP.

Besides, the DPPH-HPLC method can be used for rapid screening of antioxidants from complex mixtures, particularly for those with a minimum of sample preparation. Recently, the DPPH-HPLC method has been used to screen for free radical scavenging activity of antioxidant components in natural products, and this method has been introduced to rapidly determine the antioxidant activity of each component. Six compounds analyzed from extracts of *Eucommia ulmoides* by DPPH-HPLC exhibited the most potent inhibitory activity against DPPH radical scavenging [8]. Flavonoids from *Ginkgo biloba* and *Ampelopsis grossedentata* were identified as antioxidant compounds by the DPPH-HPLC method [10,25]. These compounds also showed real DPPH radical scavenging activities in the DPPH assay. Therefore, the DPPH-HPLC method was suggested as an effective, rapid, and easy method for the discovery of antioxidant components in TP.

*Tanacetum parthenium* L. Schultz-Bip (TP, Asteraceae) has been known for centuries as a medicinal and ornamental plant. It is native to Eurasia, specifically the Balkan Peninsula, Anatolia and the Caucasus, but cultivation has spread it around the world and it is now also found in the rest of Europe, North America and Peru [26]. TP is a medicinal plant traditionally used for the treatment of migraine headaches, rheumatoid arthritis and stomach aches [27]. In addition, TP has a long history of use in traditional and folk medicine and it has multiple pharmacologic properties, such as anticancer, anti-inflammatory, cardiotonic, antispasmodic, an emmenagogue [28]. The known chemical constituents in TP are reported to include 3β-hydroxy parthenolide, canin and artecanin, having α-methylene butyrolactone moiety [29]. However, no data have been published on the inhibitory activities of TP toward RLAR, AGEs, sorbitol accumulation, and DPPH radical scavenging regulation. Flavonoids obtained from natural extracts were reported to have strong AR inhibitory activity and may improve symptoms associated with diabetic complications. In addition, many structural properties of their type in flavonoids that inhibit RLAR have been reported [30]. The flavonoids (compounds **2**–**7**) derived from TP exhibited a wide range of inhibition. Compound **2**, **4**, and **6** are aglycone-type compounds, compounds **5** and **7** are of the methoxy aglycone type, and 3 is of the glucoside type. Upon comparing the inhibitory activities of the isolated compounds in terms of their structures, flavonol (compound **6**) showed higher RLAR inhibitory activities than flavone (compounds **2** and **4**) and methoxy flavone (compounds **5** and **7**). Furthermore, the di-hydroxy group in the C-ring of flavones, including methoxy flavones (compounds **3**, **4**, and **5**), resulted in higher RLAR inhibitory activities than mono-hydroxy and mono-methoxy groups on the same positions of the flavone C-ring. Upon comparing the inhibitory activities of compounds **3** and **4**, it seemed that increasing the number of glucosides on the A-ring would increase the inhibitory activity. Like this, a possible mechanism by which flavonoids inhibits RLAR could be related to its structure’s action position [31]. A recent study reported that compounds **2**, **4**, and **6** isolated from *Artermisia montana* and *Sophora flavescens* showed inhibitory effects on RLAR [32,33]. Compound **3** isolated from *Colocasia esculenta* displayed therapeutic potential in the prevention and treatment of diabetic complications by inhibiting RLAR activity [26]. However, the mechanism of TP and its constituents on the inhibitory effects of AR and AGEs formation have not yet been found. Therefore, TP′s physiological studies will need more for the development of phytomedicine and functional food sources.

## 4. Materials and Methods

### 4.1. Chemicals and Reagents

dl-Glyceraldehyde, the reduced form of nicotinamide adenine dinucleotide phosphate (NADPH), bovine serum albumin (BSA), sodium phosphate, methyl glyoxal, quercetin, DPPH, l-ascorbic acid, aminoguanidine, and methanol (MeOH) used in this study were purchased from Sigma (St. Louis, MO, USA). human recombinant aldose reductase (HRAR) was purchased from Wako Pure Chemical Industries (Osaka, Japan). All other chemicals and reagents used were of analytical grade.

### 4.2. Nuclear Magnetic Resonance and Mass Spectrometry Analysis

^1^H and ^13^C NMR spectra and correlation NMR spectra such as correlation spectroscopy (COSY), heteronuclear multiple bond correlation (HMBC) and heteronuclear multiple quantum coherence (HSQC) were obtained from an Avance DPX 600 spectrometer (Bruker, Madison, WI, USA). These were obtained at operating frequencies 400 (^1^H) and 100 MHz (^13^C) with CD_3_OD and TMS were used as internal standards; chemical shifts were reported in δ values. The molecular mass was measured using the low-resolution electronic impact (EI) mass spectrometer equipped JMS-700 (Tokyo, Japan). The low-resolution mass spectrometer was operated in the negative ion mode with ion source at 250 °C and EI at 70 eV with direct insertion probe and the mass range in 50–600 *m/z*. Fast atom bombardment (FAB) mass spectrometer was recorded in the negative form using *m*-nitrobenzyl alcohol as a matrix in a JEOL JMSAX 505-WA spectrometer (Tokyo, Japan).

### 4.3. Plant Materials

All 22 selected dried Peruvian plants were obtained from local markets or farms in the department of La Libertad in Peru during May–Oct. 2015. The detail vouchers were deposited at the Center for Efficacy Assessment and Development of Functional Foods and Drugs, Hallym University (Table 1). The specimens were authenticated by Paul H. Gonzales Arce in the Museo de Historia Natural Universidad Nacional Mayor de San Marcos, Lima, Peru.

### 4.4. Extraction and Isolation

The 22 dried Peruvian plants (100 g of each) were extracted with 70% MeOH (1 L × 2) for 2 h at room temperature. The combined filtrates were concentrated to dryness in vacuo at 40 °C. The extraction yields were calculated as a percentage of the dry weight of the parts used. The major components from TP were isolated by column chromatography. LNP-23 (TP, 1 g) was further purified by using a Sephadex LH-20 column with 100% MeOH as the eluent to obtain twelve pooled fractions (TP-SFracs 1–12). Among the fractions, compounds **1** (2.5 mg) and **2** (3.2 mg) were directly obtained from TP-SFracs 3 and 5, respectively. SFracs 7–9 were separated with a Sephadex LH-20 column with 70% MeOH as the eluent to obtain compounds **3** (4.1 mg), 4 (5.2 mg), and 5 (13.5 mg). TP-SFracs 11 and 12 were separated with a Sephadex LH-20 column with acetone as the eluent to obtain compounds **6** (11.7 mg) and 7 (1.6 mg).

Compound (**1**). EI-MS m/z 195 [M + H]^+^. ^1^H-NMR (400 MHz, CD_3_OD, δ_H_) δ 7.59 (1H, d, J = 15.87 Hz, H-7), 7.39 (1H, d, J = 2.02 Hz, H-2), 7.20 (1H, dd, J = 8.14, 2.02 Hz, H-6), 6.97 (1H, d, J = 8.13 Hz, H-5), 6.52 (1H, d, J = 15.86 Hz, H-8). ^13^C-NMR (100 MHz, CD_3_OD, δ_c_) δ 170.5 (C-9), 149.87 (C-4), 148.34 (C-3), 145.13 (C-7), 124.97 (C-1), 122.74 (C-6), 115.27 (C-8), 114.09 (C-5), 110.28 (C-2), 57.06 (-OCH_3_)

Compound (**2**). EI-MS *m/z* 271 [M + H]^+^. ^1^H-NMR (400 MHz, CD_3_OD, δ_H_) δ 7.87 (2H, d, *J* = 8.18 Hz, H-2′/H-6′), 6.99 (2H, d, *J* = 8.17 Hz, H-3′/H-5′), 6.75 (1H, s, H-3), 6.53 (1H, d, *J* = 2.11 Hz, H-8), 6.41 (1H, *J* = 2.11 Hz, H-6). ^13^C-NMR (100 MHz, CD_3_OD, δ_c_) δ 181.1 (C-4), 166.8 (C-7), 163.6 (C-2), 161.1(C-5), 159.7 (C-4′), 157.6 (C-9), 129.1 (C-2′/C-6′), 121.7 (C-1′), 116.4 (C-3′/C-5′), 103.7 (C-3), 102.9 (C-10), 98.8 (C-6), 94.9 (C-8).

Compound (**3**). FAB-MS *m/z* 449 [M + H]^+^. ^1^H-NMR (400 MHz, CD_3_OD, δ_H_) 7.51 (1H, dd, *J* = 8.12, 2.02 Hz, H-6′), 7.40 (1H, d, *J* = 2.04 Hz, H-2′), 6.84 (1H, d, *J* = 8.12 Hz, H-5′), 6.82 (1H, s, H-3), 6.77 (1H, d, *J* = 2.01 Hz, H-8), 6.59 (1H, d, *J* = 2.01 Hz, H-6), 5.11 (1H, d, *J* = 7.32 Hz, H-1ʹʹ), 3.85-3.36 (6H, m, H-2′′, 3 ′′, 4′′, 5′′ and 6ab′′). ^13^C-NMR (100 MHz, CD_3_OD, δ_c_) δ 181.2 (C-4), 166.1 (C-7), 164.1 (C-2), 162.0 (C-5), 157.4 (C-9), 152.7 (C-4′), 147.7 (C-5′), 126.1 (C-1′), 122.0 (C-2′), 118.1 (C-3′), 115.2 (C-6′), 105.5 (C-10), 101.2 (C-3), 101.2 (C-1′′), 98.7 (C-6), 96.7 (C-8), 76.4 (C-3′′), 75.3 (C-5′′), 74.1 (C-2′′), 72.1 (C-4′′), 65.4 (C-6′′).

Compound (**4**). EI-MS *m/z* 287 [M + H]^+^. ^1^H-NMR (400 MHz, CD_3_OD, δ_H_) δ 7.49 (1H, dd, *J* = 9.14, 1.91 Hz, H-6′), 7.29 (1H, d, *J* = 1.92 Hz, H-2′), 6.83 (1H, d, *J* = 9.17 Hz, H-5′), 6.71 (1H, s, H-3), 6.52 (1H, d, *J* = 2.00 Hz, H-8), 6.21 (1H, d, *J* = 1.99Hz, H-6). ^13^C-NMR (100 MHz, CD_3_OD, δ_c_) δ 180.3 (C-4), 166.7 (C-7), 164.5 (C-2), 160.7 (C-5), 158.5 (C-9), 148.5 (C-4′), 145.0 (C-5′), 120.5 (C-1′), 118.7 (C-2′), 116.8 (C-3′), 113.1 (C-6′), 103.2 (C-3), 101.7 (C-10), 99.1 (C-6), 96.9 (C-8).

Compound (**5**). EI-MS *m/z* 361 [M + H]^+^. ^1^H-NMR (400 MHz, CD_3_OD, δ_H_) δ 7.56 (1H, dd, *J* = 9.13, 2.02 Hz, H-6′), 7.31(1H, d, *J* = 1.99 Hz, H-2′), 6.87 (1H, d, *J* = 9.12 Hz, H-5′), 6.53 (1H, s, H-8), 3.81 (9H, m, H-3, 6 and 7, OCH_3_). ^13^C-NMR (100 MHz, CD_3_OD, δ_c_) δ 178.6 (C-4), 159.7 (C-7), 156.2 (C-9), 153.2 (C-2), 151.3 (C-5), 147.2 (C-4′), 145.9 (C-3′), 138.5 (C-3), 133.7 (C-6), 123.8 (C-1′), 120.3 (C-6′), 118.2 (C-5′), 115.9 (C-2′), 107.9 (C-10), 95.8 (C-8), 60.8 (C-6, OCH_3_), 58.6 (C-3, OCH_3_), 56.1 (C-7, OCH_3_).

Compound (**6**). EI-MS *m/z* 287 [M + H]^+^. ^1^H-NMR (400 MHz, CD_3_OD, δ_H_) δ 7.84 (2H, d, *J* = 8.17 Hz, H-2′/H-6′), 6.94 (2H, d, *J* = 8.15 Hz, H-3′/H-5′), 6.54 (1H, d, *J* = 2.03 Hz, H-8), 6.42 (1H, *J* = 2.07 Hz, H-6). ^13^C-NMR (100 MHz, CD_3_OD, δ_c_) δ 177.1 (C-4), 165.7 (C-7), 164.2 (C-5), 161.2 (C-9), 157.8 (C-4′), 148.6 (C-2), 136.5 (C-3), 128.6 (C-2′/C-6′), 121.9 (C-1′), 117.7 (C-3′/C-5′), 101.3 (C-10), 98.1 (C-6), 95.3 (C-8).

Compound (**7**). EI-MS *m/z* 361 [M + H]^+^. ^1^H-NMR (400 MHz, CD_3_OD, δ_H_) δ 7.83 (2H, d, *J* = 8.09 Hz, H-2′/H-6′), 7.03 (2H, d, *J* = 8.07 Hz, H-3′/H-5′), 6.59 (1H, s, H-8), 3.87 (9H, m, H-3, 6 and 4′, OCH_3_). ^13^C-NMR (100 MHz, CD_3_OD, δ_c_) δ 179.2 (C-4), 160.8 (C-4′), 159.3 (C-7), 157.8 (C-9), 156.9 (C-2), 153.4 (C-5), 141.2 (C-3), 132.9 (C-6), 129.3 (C-2′/6′), 121.7 (C-1′), 116.7 (C-3′/5′), 107.2 (C-10), 96.6 (C-8), 61.1 (C-6, OCH_3_), 59.4 (C-3, OCH_3_), 57.1 (C-4′, OCH_3_).

### 4.5. High-performance Liquid Chromatography Analysis

HPLC analysis was performed on an Agilent 1100 series system equipped with a diode-array detector (DAD, Agilent, Sunnyvale, CA, USA), consisting of a vacuum degasser (G1322A), a quaternary pump (G1311A), an autosampler (G1313A), a thermostatted column compartment (G1316A), and a DAD (G1315B) system. Separation was achieved on an Eclipse XDB-phenyl column (150 mm × 4.6 mm, 3.5 μm), coupled with a guard column, at 30 °C. Samples (10 µL) were injected into the system. The samples were eluted with acidified water (0.1% trifluoroacetic acid, A) and MeOH (B), at a flow rate of 0.7 mL/min. The optimized gradient conditions were as follows: 5%–100% B at 0–50 min; 100%–5% B at 50–55 min; isocratic 5% B at 55–60 min. The detector monitored the eluent at a wavelength of 254 nm.

### 4.6. Human Recombinant Aldose Reductase Ultrafiltration High-performance Liquid Chromatography Assay

The inhibitory compounds of HRAR from TP were profiled by an AR ultrafiltration assay. Specifically, TP (at a final concentration of 0.1 mg/mL) was incubated with 0.6 M ammonium sulfate and 3.9 μM HRAR in a total volume of 300 μL at 37 °C for 30 min. Then, the incubated mixture was filtered by using a Microcon YM-10 centrifugal filter unit by centrifugation at 5167× *g* for 30 min at room temperature. The filtrate was subsequently analyzed by using the methods mentioned in the HPLC analysis section. A sample incubated without HRAR was used as a control. The relative binding affinity of the inhibitors from TP toward HRAR was defined as the “binding degree” (BD), which can be calculated as follows: BD (%) = (A_a_ − A_b_)/A_a_ × 100, in which A_a_ and A_b_ are the peak areas of a compound without and with HRAR in the HPLC chromatograms, respectively [34].

### 4.7. Preparation of Rat Lens Aldose Reductase

Crude RLAR was prepared as follows: Crude RLAR was prepared as follows: lenses were removed from Sprague-Dawley rats (Weighing 250–280 g) and frozen at −70 °C until use. Non-cataractous transparent lenses were pooled, and a homogenate was prepared in 0.1 M phosphate-buffered saline (pH 6.2). The RLAR homogenate was then centrifuged at 10,000× *g*for 20 min at 4 °C in a refrigerated centrifuge. The supernatant was collected and used as the RLAR [35]. This experiment was approved by the University of Hallym Animal Care and Use Committee (Registration Number: Hallym R2016-60). All of the procedures were conducted in accordance with the ‘Guide for Care and Use of Laboratory Animals’, published by the National Institutes of Health.

### 4.8. Determination of Rat Lens Aldose Reductase Inhibition

A total of 531 μL of 0.1 M potassium buffer (pH 7.0), 90 μL of NADPH solution (1.6 mM in potassium buffer), 90 μL of RLAR homogenate (6.5 U/mg), 90 μL of ammonium sulfate solution (4 M in potassium buffer), and 90 μL of DL-glyceraldehyde (25 mM in potassium buffer) were mixed with 9 μL of different concentrations of samples (1–0.1 mg/mL in dimethylsulfoxide (DMSO), Less than 1% in total mixture) in a cuvette, and the activity of RLAR was assessed spectrophotometrically by measuring the decrease in NADPH absorbance at 340 nm for 3 min using a spectrophotometer (SECOMAM, Ales Cedex, France). Quercetin was used as positive controls. The inhibition of RLAR (%) was calculated using the following equation: (1 − (△A sample/min) – (△A blank/min)/(△A control/min) − (△A blank/min)) × 100%, where △A sample/min is the decrease in absorbance over 3 min with reaction solution, test sample, and substrate, and △A control/min is the same but with DMSO (Less than 1% in total mixture) instead of test sample [36].

### 4.9. Lens Culture and Intracellular Sorbitol Measurement

Lenses isolated from 10-week-old Sprague-Dawley rats were cultured for 6 d in TC-199 medium that contained 15% fetal bovine serum, 100 units/mL penicillin, and 0.1 mg/mL streptomycin, under sterile conditions and an atmosphere of 5% CO_2_ and 95% air at 37 °C. The lenses were divided into three groups and cultured in medium containing 30 mM glucose and active compounds. Each lens was placed in a well containing 2.0 mL of medium. Sorbitol was determined by HPLC after derivatization by reaction with benzoic acid to a form of a fluorescent compound [37].

### 4.10. DPPH High-performance Liquid Chromatography Assay

The antioxidant compounds from TP were profiled with a DPPH-HPLC assay. TP (90 µL; 10.0 mg/mL) was mixed with 540 µL of DPPH solution (0.32 mM), and the mixed solution was then incubated at 37 °C for 20 min in a dark room. The solution was then filtered through a 0.45 µm membrane filter and then subjected to HPLC analysis. The HPLC conditions were the same as those described in the HPLC analysis section [38].

### 4.11. Evaluation of DPPH Radical Scavenging Capacity

The DPPH radical scavenging capacity assay method of Yin et al. was used [18]. DPPH (0.32 mM) was diluted with MeOH, and this solution (180 µL) was mixed with 30 µL of the sample at each concentration. After 20 min of incubation in a darkroom, the absorbance at 570 nm was recorded on a microplate reader (EL800 Universal Microplate reader, Bio-Tek Instruments, Winooski, VT, USA). DPPH radical scavenging activity was expressed as the percentage inhibition (%) of DPPH in the aforementioned assay system and was calculated as (1 – B/A) × 100, in which A and B are the activities of DPPH without and with the test material, respectively.

### 4.12. Bovine Serum Albumin–Methylglyoxal Assay for Advanced Glycation End Products Formation

BSA (50 mg/mL) was incubated with methylglyoxal (100 mM) in sodium phosphate buffer (0.1 M, pH 7.4) in the presence of various concentrations of the compounds (including a control) at 37 °C for 24 h. Then, the fluorescence intensity was measured at an excitation wavelength of 355 nm and an emission wavelength of 460 nm with a luminescence spectrometer (LS50B, Perkin–Elmer Ltd., Buckinghamshire, UK). The DMSO used as a vehicle was found to have no effect on the reaction. All reagents and samples were sterilized by filtration through 0.45 mm membrane filters [39].

### 4.13. Statistical Analysis

Inhibition rates were calculated as percentages (%) with respect to the control value, and the IC_50_ value was defined as the concentration at which 50% inhibition occurred. Data are expressed as mean values ± standard deviation of triplicate experiments.

## 5. Conclusions

In summary, RLAR and DPPH radical scavenging of 22 Peruvian plant extracts were investigated. TP, which showed the highest inhibitory activities towards RLAR, AGEs formation, and DPPH radical scavenging, and considerable anti-diabetic complication effects, would be a good ingredient for the development of a functional material. Furthermore, the ultrafiltration technique using RLAR and DPPH-HPLC radical scavenging detection system, which facilitates the rapid determination of active components in a natural product, revealed the presence of the six active components in TP. Sephadex LH-20 column chromatography was successfully applied to separate and purify these compounds, which were identified as ferulic acid (**1**), apigenin (**2**), luteolin-7-*O*-glucoside (**3**), luteolin (**4**), chrysosplenol (**5**), kampferol (**6**), and santin (**7**). These compounds showed strong inhibitory activities against RLAR, including sorbitol accumulation, the formation of advanced glycation, and DPPH radical scavenging. Consequently, we conclude that TP and its constituents can be used as natural drugs and functional food sources to treat diabetic complication and the RLAR ultrafiltration and DPPH-HPLC methods used herein constitute a very simple, straightforward, rapid, robust, and selective technology for discovering bioactive components of TP. Finally, this work provides a priority list of interesting plants for further study with respect to the treatment of diabetic complication and associated diseases and can be formed the scientific base of the traditional uses of the tested plants.

## Figures and Tables

**Table 1:** The Rat Lens Aldose Reductase (RLAR), 2,2-diphenyl-1-picrylhydrazyl (DPPH) radical scavenging activities, and yields of 22 Peruvian plants extracts from La Libertad.

Catalogues	Family	Spices	Common Names	Used Part	Yield (%)	Inhibition (%)	
						RLAR	DPPH
LNP-1	PTERIDACEAE	*Cheilanthes pruinata* Kaulf.	Cuti-Cuti Marron macho	Aerial part	30.3	41.6 ± 1.1	53.2 ± 0.3
LNP-3	FABACEAE	*Otholobium mexicanum* (L. f.) J.W. Grimes	Culen Negro	Aerial part	6.2	50.4 ± 0.5	4.5 ± 5.7
LNP-7	FABACEAE	*Senna* sp.	Hoja de sen	Leaf	8.2	29.6 ± 2.3	NI ^a^
LNP-11	LAMIACEAE	*Ocimum basilicum* L.	Albahaca de olor	Leaf	5.6	16.5 ± 0.8	6.0 ± 1.4
LNP-13	POACEAE	*Chrysopogon zizanioides* (L.) Roberty	Pachuli	Leaf	9.1	5.8 ± 3.4	NI
LNP-15	LAMIACEAE	*Clinopodium pulchellum* (Kunth) Govaerts	Panisara	Leaf	4.2	38.5 ± 2.5	52.8 ± 3.4
LNP-18	POACEAE	*Cymbopogon citratus* (DC.) Stapf	Hierba Luisa	Leaf	6.9	3.2 ± 0.1	2.4 ± 1.3
LNP-19	SCROPHULAR-IACEAE	*Buddleja americana* L.	Flor Blanca	Flowers	8.6	2.8± 0.2	2.8 ± 1.1
LNP-20	CARYOPHYL-LACEAE	*Dianthus caryophyllus* L.	Clavel	Leaf	12.4	23.1 ± 3.4	6.4 ± 4.8
LNP-23	ASTERACEAE	*Tanacetum parthenium* (L.) Sch. Bip.	Santa Maria	Whole	10.3	61.1 ± 0.5	88.6 ± 2.1
LNP-24	CAPRIFOLIAEAE	*Sambucus peruviana* H. B. K	Sauco (tilo)	Leaf	8.9	51.3 ± 0.9	53.3 ± 1.6
LNP-27	LYCOPODIACE-AE	*Huperzia crassa* (Humb. & Bonpl. ex Willd.) Rothm.	Trensilla or enredadera	Leaf	12.3	19.7 ± 0.9	NI
LNP-28	CYPERACEAE	*Eleocharis albibracteata* Nees & Meyen ex Kunth	Hierba del caballero	Leaf	3.9	50.2 ± 1.4	23.6 ± 2.3
LNP-33	ASTERACEAE	*Werneria nubigena* Kunth	Condor	Leaf	8.6	43.9 ± 1.8	11.4 ± 7.0
LNP-39	EUPHORBIACE-AE	*Jatropha curcas* L.	Pinones, pinol	Seed	2.0	16.2 ± 3.2	4.3 ± 9.6
LNP-41	ANACARDIAC-EAE	*Anacardium occidentale* L.	Pepa de la selva, Casho	Fruit	25.7	16.2 ± 0.2	36.7 ± 0.4
LNP-43	LAURACEAE	cf. *Endlicheria*	Spingo	Seed	10.1	3.3 ± 0.4	2.3 ± 0.9
LNP-45	SOLANACEAE	*Capsicum chinense*	Aji panca rojo	Fruit	42.0	2.1 ± 0.3	4.1 ± 0.3
LNP-46	SOLANACEAE	*Capsicum baccatum*	Aji amarillo	Fruit	50.9	8.7 ± 0.7	1.1 ± 0.2
LNP-47	FABACEAE	*Lupinus mutabilis*	Tarwi	Seed	10.2	NI	NI
LNP-48	URTICACEAE	*Urtica magellanica* A. Jussie ex Poiret	Ortiga Negra	Aerial part	6.6	NI	4.3 ± 6.9
LNP-80	FABACEAE	*Desmodium molliculum* (Kunth) DC.	Manayupa	Leaf	23.8	12.9 ± 0.5	90.0 ± 4.8

^a^ NI is not inhibition.

**Table 2:** The inhibitory effects of *Tanacetum parthenium* L. extract on RLAR, DPPH radical scavenging activity, and advanced glycation end products (AGEs).

Entry		Concentrations (μg/mL)	Inhibition (%)	IC_50_ (μg/mL) ^a^
RLAR	70% MeOH	10	61.10	8.04 ± 0.61
		5	31.24	
		1	20.35	
	Quercetin ^b^	10	81.28	5.35 ± 0.20
		1	44.80	
		0.5	23.56	
DPPH	70% MeOH	75	88.58	33.22 ± 2.09
		30	49.53	
		15	31.84	
	L-ascorbic acid ^c^	15	97.23	6.02 ± 0.37
		7.5	58.34	
		3	33.71	
AGEs	70% MeOH	200	61.01	163.71 ± 6.31
		100	30.49	
		20	8.16	
	Aminoguanidine ^d^	200	75.32	121.96 ± 5.10
		100	39.94	
		20	20.75	

^a^ The IC_50_ value was defined as a mean ± SEM of the half-maximal inhibitory concentration of the results obtained from three independent experiments performed in duplicate. ^b–d^ Quercetin, L-ascorbic acid, and aminoguanidine are the positive controls for RLAR, DPPH and AGEs assay, respectively.

**Table 3:** Inhibitory effects of compounds isolated from *Tanacetum parthenium* L. on RLAR, DPPH radical scavenging activity, and AGEs.

Compounds	RLAR		DPPH		AGEs
	IC_50_ (μM) ^a^	TBD (%) ^b^	IC_50_ (μM)	PAR (%) ^c^	AGEs
Ferulic acid (**1**)	3.20 ±0.12	27.08	16.23 ± 0.41	23.53	5.59 ± 0.26
Apigenin (**2**)	1.97 ± 0.10	15.00	14.06 ± 0.72	11.28	NI
Luteolin-7-*O*-glucoside (**3**)	1.31 ± 0.09	26.58	6.44 ± 0.14	16.85	3.43 ± 0.12
Luteolin (**4**)	1.76 ± 0.03	38.02	11.84 ± 0.37	29.36	6.73 ± 0.43
Chrysosplenol (**5**)	1.92 ± 0.08	25.54	>25	7.19	NI
Kaempferol (**6**)	1.11 ± 0.03	24.29	8.32 ± 0.54	13.97	NI
Santin (**7**)	NI ^b^	-	NI	-	NI
Quercetin ^d^	1.77 ± 0.53	-	-	-	-
L-ascorbic acid ^e^	-	-	3.41 ± 0.11	-	-
Aminoguanidine ^f^	-	-	-	-	110.55 ± 3.28

^a^ The IC_50_ value was defined as a mean ± SEM of half-maximal inhibitory concentration from three independent experiments performed in duplicate and the range of the inhibitor concentrations adopted to evaluate IC_50_ was prepared as follows: 1) RLAR: 1, 5, and 10 μg/mL, 2) DPPH: 15, 30, and 75 μg/mL, 3) AGEs: 10, 25, and 50 μg/mL. ^b^ TBD is total binding degree calculated from aldose reductase ultrafiltration HPLC. ^c^ PAR is peak area reduction calculated from DPPH-HPLC. ^d–f^ Quercetin, L-ascorbic acid and aminoguanidine were the positive control for RLAR, DPPH radical scavenging and AGEs.

**Table 4:** Inhibitory effects of rat lens aldose reductase-active compounds *Tanacetum parthenium* L. on sorbitol accumulation in the rat lens.

Compounds	Sorbitol Content (mg)/lens Wet Weight (g)	Inhibition (%)
Sorbitol free	No detection	-
Control	1.47 ± 0.04	-
Quercetin ^a^	0.21 ± 0.02	85.71 ± 5.71
Ferulic acid (**1**)	0.29 ± 0.03	80.27 ± 2.38
Apigenin (**2**)	0.19 ± 0.06	87.07 ± 2.37
Luteolin-7-*O*-glucoside (**3**)	0.07 ± 0.01	95.23 ± 5.97
Luteolin (**4**)	0.12 ± 0.02	91.83 ± 5.23
Chrysosplenol (**5**)	0.26 ± 0.03	82.31 ± 2.39
Kaempferol (**6**)	0.03 ± 0.00	97.95 ± 6.31

^a^ Quercetin is the positive control for sorbitol accumulation. Results are presented as mean ± SD (*n* = 3).

## Abbreviations

AGEs	advanced glycation end products
AR	aldose reductase
ARIs	aldose reductase inhibitors
BSA	bovine serum albumin
COSY	correlation spectroscopy
DPPH	2,2-diphenyl-1-picrylhydrazyl
DMSO	dimethylsulfoxide
HMBC	heteronuclear multiple bond correlation
HPLC	high-performance liquid chromatography
HRAR	human recombinant aldose reductase
HSQC	heteronuclear multiple quantum coherence
MeOH	methanol
NADPH	nicotinamide adenine dinucleotide phosphate
NMR	nuclear magnetic resonance
PAR	peak area reduction
RLAR	rat lens aldose reductase.
TBD	total binding degree
TP	*Tanacetum parthenium*
